# Organ-Sparing Surgery for a Giant Verrucous Carcinoma of the Penile Shaft: A Case Report and Review of the Literature

**DOI:** 10.1155/2019/1537379

**Published:** 2019-02-24

**Authors:** Prodromos Philippou, Christos Kitsios, Maria Miliatou, Christiana Poullou, Pavlos Konstantinou

**Affiliations:** ^1^St George's Medical School, University of Nicosia, Nicosia, Cyprus; ^2^Apollonion Hospital, Nicosia, Cyprus; ^3^Histopathology and Cytology Laboratory Services, Nicosia, Cyprus; ^4^School of Medicine, European University of Cyprus, Cyprus

## Abstract

Verrucous Carcinoma is a rare but well-differentiated variant of penile squamous cell carcinoma. Its clinical presentation is usually that of an exophytic cauliflower-like lesion with a broad-based growth pattern. We herein report the case of a 61-year-old man who presented with a giant verrucous carcinoma occupying the dorsal surface of the penile shaft. The patient underwent penile-sparing surgery, achieving both disease control and organ preservation. We discuss relevant issues, including clinical features, diagnosis, surgical management, and prognosis and we review the rather sparse literature regarding this rare lesion.

## 1. Introduction

Penile carcinoma is a rare malignancy in the West, with an incidence of 0.1-0.9 cases per 100.000 adult males in Europe [1, 2]. A significant geographical variation of this malignancy is noted, as the incidence may be as high as 19 cases per 100.000 males in some parts of Asia, South America, and Africa [1, 2].

Verrucous carcinoma (VC) is a relatively rare and well-differentiated variant of penile squamous cell carcinoma. Its biological behavior is that of a well-differentiated tumor with an extremely low metastatic potential [3–5]. Penile VC may, however, exhibit a pattern of aggressive local growth and occasionally presents as locally advanced disease [6, 7]. Historically, in the vast majority of patients presenting with penile carcinomas, the primary lesion was managed by either radical surgery (partial or total penile amputation) or radiotherapy, but the current trend is for a less aggressive (i.e., organ-sparing) surgical approach [8–10]. In the case of VC, due to the rarity of the disease, solid data on optimal management are lacking.

We herein report the case of a 61-year-old man who presented with a large VC occupying the penile shaft and was successfully managed by penile-preserving surgery.

## 2. Case Presentation

**Figure 1:**
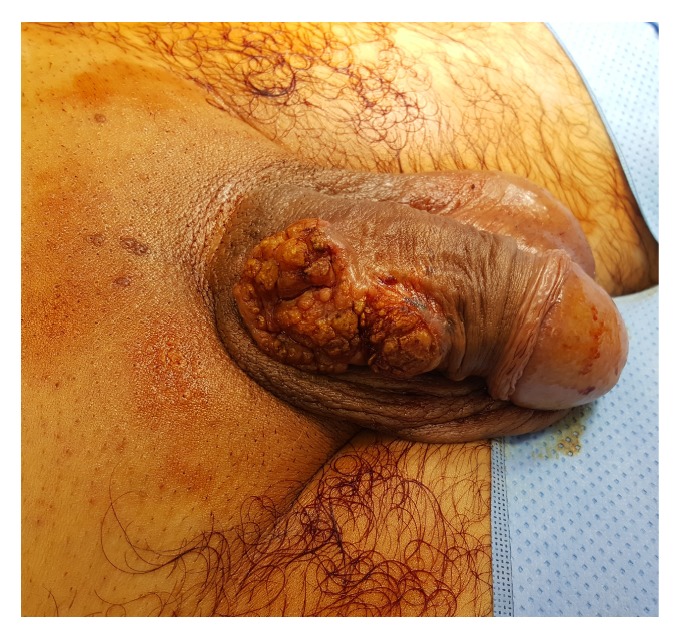
A large exophytic, cauliflower-like, and partially ulcerated lesion, measuring 7x4 cm located at the dorsal surface of the penile shaft.

A 61-year-old policeman, with no previous urological history, presented with a large exophytic, cauliflower-like, and partially ulcerated lesion, measuring 7x4 cm located at the dorsal surface of the penile shaft (Figure 1). The patient reported a past medical history of hypertension but was otherwise fit and healthy. He was sexually active, denied erectile dysfunction, and had never smoked. On palpation, the lesion was large but mobile and did not appear to invade the corpora cavernosa. Physical examination confirmed the absence of palpable inguinal nodes, and routine laboratory tests (including Alkaline Phosphatase and Calcium levels) were within normal limits. After excluding infections, including sexually transmitted diseases, an incisional biopsy of the lesion revealed the presence of low-grade squamous cell carcinoma and the absence of lymphovascular invasion. Staging CT of the chest, abdomen, and pelvis did not demonstrate metastatic disease or enlarged lymph nodes.

**Figure 2:**
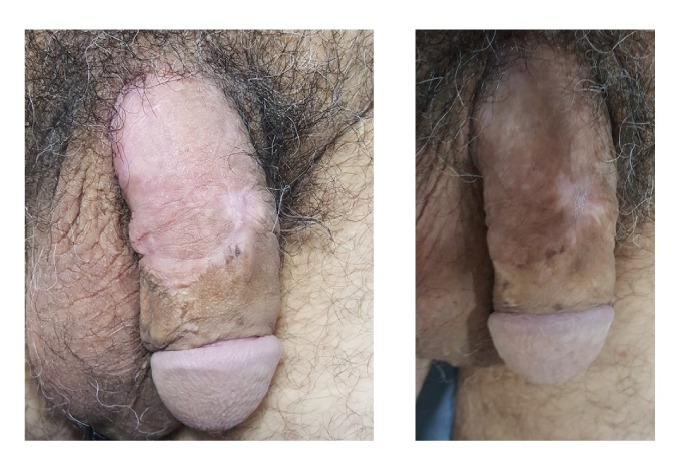
A satisfactory cosmetic result was recorded at 3 months (left) and 6 months (right) postoperatively.

After discussion, further surgical management was decided, including excision of the primary lesion and reconstruction of the tissue gap. The patient underwent wide local excision of the affected area of skin in the penile shaft, and intraoperative frozen section analysis was used to confirm margin negativity. A circumcision was also performed, and the penis was grafted with a partial-thickness skin graft. In particular, a 0.4 mm split-skin graft was harvested from the lateral thigh with an air dermatome and was used to cover the tissue defect. The graft was quilted using 5–0 interrupted polyglactin sutures. The patient made an excellent recovery with resolution of symptoms and restoration of sexual function. A satisfactory cosmetic result was recorded at 3 and 6 months postoperatively (Figure 2).

**Figure 3:**
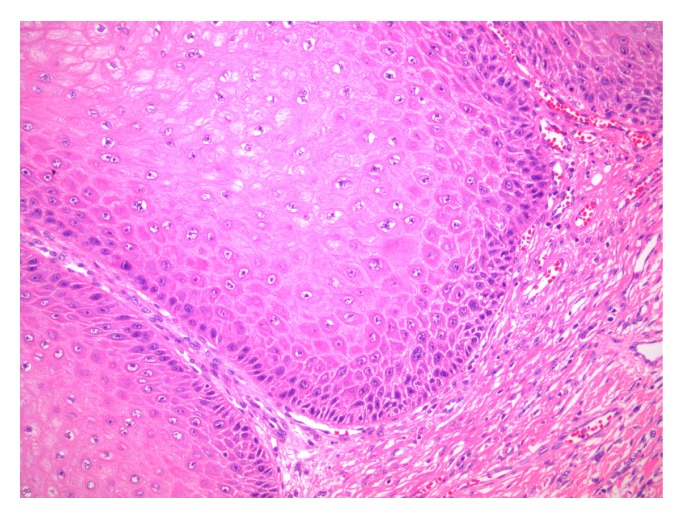
On microscopy, the tumor front is broad-based and pushes the subepithelial tissues [haematoxylin-eosin stain, original magnification x200].

**Figure 4:**
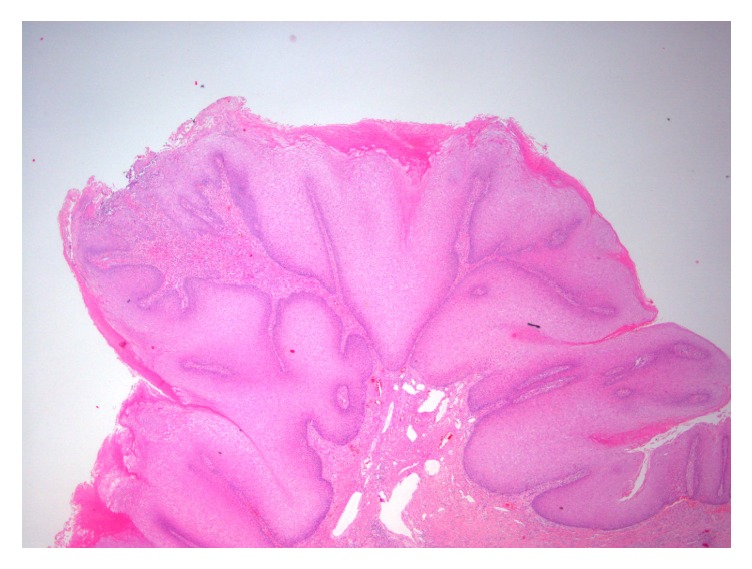
The tumor is characterized by prominent papillomatosis and acanthosis [haematoxylin-eosin stain, original magnification x20].

Histological examination confirmed the presence of a very well-differentiated neoplasm with sharp delineation at the tumor-stroma junction. On microscopy, the tumor exhibited hyperkeratosis, papillomatosis, and acanthosis. The tumor front was broad-based and pushed the subepithelial tissues. Small foci of clear-cut infiltration of subepidermal connective tissue, however, were also present. The neoplastic cells were well-differentiated, with minimal deviation from normal squamous cells and small, bland, round, or vesicular nuclei. Cellular atypia was focal and minimal, while mitoses were rare (Figures 3 and 4). The morphological findings were consistent with verrucous carcinoma of the penis. Lymphovascular invasion or perineural invasion was not identified (pT1a disease).

Based on final histology, the patient was classified as low risk for nodal involvement and was managed with surveillance for recurrence at the primary site and the groin areas as per current guidelines. Clinical examination and follow-up imaging up to 3 years postoperatively showed no locoregional recurrence or distant metastases.

## 3. Discussion

In 1925, Buschke and Löwenstein described ‘carcinoma-like condylomata acuminata' as a locally invasive, rapidly growing tumor [11]. Buschke-Löwenstein tumor is classified as a verrucous carcinoma. The term ‘verrucous carcinoma' was first introduced by Ackerman in 1948 and has been reported in the oral cavity, anus, penis, and female genitalia [12]. Buschke-Löwenstein tumor is generally considered as verrucous carcinoma involving the genital regions, but in some reports the lesions are regarded as distinct entities [12, 13]. It is a less common variant of penile squamous cell carcinoma, accounting for 3-8% of all penile tumors [13].

The exact etiology of verrucous carcinoma is yet to be clarified. Human Papilloma Virus types 6 and 11 have been linked with the pathogenesis of this tumor, but other studies have shown that HPV infection is not a universal finding in VC [5]. Risk factors for VC are low socioeconomic status, drug abuse, sexually transmitted diseases, diabetes, and smoking [12]. A mechanism related to impaired immune response has been proposed, and anogenital VC in association with HIV infection has been described [14].

Macroscopically, it is challenging to identify penile VC due to the gross similarities with condyloma acuminatum. It usually involves the glans as the common squamous cell carcinoma and presents as a cauliflower- or wart-like painless lesion. Verrucous carcinoma, however, grows slowly and may invade the glans or even the shaft. If left untreated, certain verrucous carcinomas evolve to giant masses which may be foul-smelling, painful, and ulcerated [7].

The literature on verrucous carcinoma mostly focuses on case reports and rarely on large-scale studies. Nevertheless, surgical treatment for penile verrucous carcinoma has been generally accepted as the mainstay for treatment. Since penile VC exhibits nonaggressive biological behavior, organ-sparing surgery, if possible, is an acceptable treatment option to avoid mutilating surgery and maintain the appearance and function of the penis [3, 8, 10]. VC, a well-differentiated tumor, is ideal for a wide range of conservative excisional and reconstructive techniques. A low threshold for intraoperative frozen section analysis of excision margins is recommended to balance oncological safety and preservation of functional penile length, especially if the macroscopic margin is under question [9, 10]. Despite the lack of metastatic potential, the chance of local recurrence is not to be overlooked, but rates vary between studies and appear to be significantly lower compared to the aggressive perianal VC [7, 15].

The optimal technique for penile skin reconstruction remains a point of debate and appropriate selection of graft type depends on the size and location of the defect. Some surgeons prefer full-thickness skin grafts, due to their greater elasticity and less primary contraction after harvesting. Split-thickness skin grafts, however, require less ideal conditions for survival and have lower incidence of graft failure. We apply the latter, harvested from the thigh in order to minimise donor-site morbidity and to achieve adequate cosmetic and functional results [8, 16].

Systemic or intralesional chemotherapy (with 5-fluorouracil or cisplatin) and intralesional interferon have been suggested, as alternative monotherapies or as an adjunct to surgery [6, 7]. Intra-aortic infusion chemotherapy (with methotrexate or mitomycin C plus 5-fluorouracil) has been used in a small series of patients with advanced penile VC and the results appear promising but large-scale data are lacking [6]. Radiotherapy remains controversial, due to the concern for radiation-induced anaplastic transformation of the primary verrucous carcinoma [6, 7]. Carbon dioxide laser ablation has also been tried with positive results in younger patients with smaller lesions confined to the glans [17, 18].

According to the limited amount of available evidence, local excision of VC with negative margins results in favorable long-term prognosis [19]. In previous case series, inguinal lymphadenectomy was performed in some patients but there was no nodal involvement [3, 15, 19]. Thus, inguinal lymphadenectomy is not recommended. One should note, however, that according to the findings of Shimizu et al. [20], foci of invasive squamous cell carcinoma were found in ~30% of verrucous carcinomas and the possibility of progression to other types of invasive squamous cell carcinoma is not to be excluded. This finding is relevant in the context of counselling and close follow-up of patients who undergo penile-conserving surgery. In the case presented herein, the presence of subepithelial invasion (pT1a disease) is of concern in regard to the increased risk of local recurrence [10]. Since local recurrence is more likely after organ-sparing surgery, commitment to a strict follow-up schedule according to the EAU guidelines should be ensured [1].

## 4. Conclusion

Penile verrucous carcinoma is a rare clinical entity with distinct clinical and histological characteristics. Organ-sparing excisional surgery with appropriate reconstruction achieves adequate disease control with excellent cosmesis. A well-structured approach, based on clinical and histological features, is essential, to avoid possible diagnostic pitfalls and overtreatment. The lack of clear recommendations based on evidence, however, underscores the need for multicenter studies with adequate sample size and follow-up.
